# Differences in Coping Strategies and Help-Seeking Behaviours among Australian Junior and Senior Doctors during the COVID-19 Pandemic

**DOI:** 10.3390/ijerph182413275

**Published:** 2021-12-16

**Authors:** Amy Pascoe, Eldho Paul, Douglas Johnson, Mark Putland, Karen Willis, Natasha Smallwood

**Affiliations:** 1 Department of Allergy, Immunology and Respiratory Medicine, Central Clinical School, The Alfred Hospital, Monash University, Melbourne 3004, Australia; amy.pascoe@svha.org.au; 2 Department of Epidemiology and Preventive Medicine, School of Public Health and Preventive Medicine, Monash University, Melbourne 3004, Australia; eldho.paul@monash.edu; 3 Clinical Haematology Department, The Alfred Hospital, Melbourne 3004, Australia; 4 Departments of General Medicine and Infectious Diseases, Royal Melbourne Hospital, Parkville 3050, Australia; douglas.f.johnson@mh.org.au; 5 Department of Medicine, Royal Melbourne Hospital, University of Melbourne, Parkville 3050, Australia; 6 Department of Emergency Services, Royal Melbourne Hospital, Grattan Street, Parkville 3050, Australia; mark.putland@mh.org.au; 7 Department of Critical Care, Faculty of Medicine Dentistry and Health Sciences, University of Melbourne, Parkville 3052, Australia; 8 School of Public Health, College of Health and Biomedicine, Victoria University, Melbourne 3011, Australia; karen.willis@vu.edu.au; 9 Division of Critical Care and Investigative Services, Royal Melbourne Hospital, Grattan Street, Parkville 3050, Australia; 10 Department of Respiratory Medicine, The Alfred Hospital, 55 Commercial Road, Prahran 3004, Australia

**Keywords:** COVID-19, coping, mental health, doctors, frontline

## Abstract

Background: Throughout the COVID-19 pandemic, hospital medical staff (HMS) have faced significant personal, workplace, and financial disruption. Many have experienced psychosocial burden, exceeding already concerning baseline levels. This study examines the types and predictors of coping strategies and help-seeking behaviours utilised by Australian junior and senior HMS during the first year of the pandemic. Methods: A cross-sectional online survey of Australian frontline healthcare workers was conducted between 27 August and 23 October 2020. Data collected included demographics, personal and workplace disruptions, self-reported and validated mental health symptoms, coping strategies, and help-seeking. Results: The 9518 participants included 1966 hospital medical staff (62.1% senior, 37.9% junior). Both groups experienced a high burden of anxiety, depression, post-traumatic stress disorder, and burnout. Coping strategies varied by seniority, with maintaining exercise the most common strategy for both groups. Adverse mental health was associated with increased alcohol consumption. Engagement with professional support, although more frequent among junior staff, was uncommon in both groups. Conclusions: Junior and senior staff utilised different coping and help-seeking behaviours. Despite recognition of symptoms, very few HMS engaged formal support. The varied predictors of coping and help-seeking identified may inform targeted interventions to support these cohorts in current and future crises.

## 1. Introduction

Workplace stressors and mental health problems are recognised issues for hospital doctors [1,2,3,4,5]. The severe acute respiratory syndrome coronavirus 2 (SARS-CoV-2 or COVID-19) pandemic and associated public health restrictions have compounded these issues, resulting in significant workplace, social and financial disruption, moral distress, and mental health symptoms for frontline healthcare workers in Australia and internationally [6,7,8,9,10,11,12]. Adaptation to repeated and sometimes sudden changes in workload, work roles, PPE and visitor guidelines, and to alternative delivery models such as telehealth, have been required, often with inadequate communication or support from the workplace [7].

A growing body of work has investigated the specific types of coping strategies utilised by doctors during crises, including the severe acute respiratory syndrome (SARS) pandemic in 2003 [13], natural disasters [14], and more recently, COVID-19 [10,15,16]. Adopting effective positive coping strategies, such as engaging with social supports and maintaining physical exercise, during and after a crisis can mediate the impacts of crisis events on mental health [17,18]. Conversely, relying on negative coping strategies, particularly increasing alcohol consumption, can have lasting detrimental effects even after the initial crisis has resolved [13] and is associated with an increased risk of burnout [19].

Pre-pandemic survey data from 2013 identified that mental illnesses affected one third of Australian junior doctors and one quarter of consultants [20]. Burnout symptoms were common, with up to 45% of interns and trainees and 28% of consultants experiencing emotional exhaustion [20,21,22]. Despite this high burden of mental health symptoms, doctors are known to be reluctant either to discuss mental health issues or to seek professional help for them [21,23,24]. This reluctance is particularly prevalent among junior doctors. For example, 68.4% of Irish junior doctors experienced self-stigma [22], and Australian junior doctors worried that disclosing mental health concerns may have detrimentally affected their career prospects [24].

Since the onset of the COVID-19 pandemic, many professional associations have emphasised the need for clinicians to seek support for mental health [25,26,27]. Despite this, only one in ten UK physicians have reported seeking formal support [26]. Understanding the types of coping strategies and help-seeking behaviours utilised by junior and senior hospital medical staff, and how these behaviours vary during a crisis, can inform productive interventions to safeguard this workforce.

The Australian COVID-19 Frontline Healthcare Workers Study is an initiative led by frontline clinicians in partnership with academics to investigate the mental health impacts of the COVID-19 pandemic on Australian healthcare workers and to quantify the prevalence and severity of workplace, social, and financial disruptions during it [6]. Previous findings from this study identified that, despite a high burden of symptoms, healthcare workers utilised a range of coping strategies largely influenced by demographic factors, with very few of them seeking professional support [10].

It is unclear how the additional disruptions and stress associated with COVID-19 have differentially impacted the coping strategies and help-seeking behaviours of junior and senior doctors. We hypothesised that the types and predictors of coping strategies and help-seeking behaviours would vary according to the seniority of doctors. This paper reports a subset of our findings. It aims to describe the types of coping strategies and help-seeking behaviours utilised by Australian junior and senior doctors, and to identify the personal and professional predictors of adoption of these.

## 2. Materials and Methods

### 2.1. Study Design and Sample

The full study methodology has been published [6]. In summary, a nationwide, voluntary, anonymous, online survey was conducted between 27 August and 23 October 2020, concurrent with the Australian second wave of the pandemic which occurred primarily in Melbourne, Victoria [28]. Self-identified frontline healthcare workers from all health backgrounds across Australia were invited to participate. Participants did not need to have direct contact with people infected with COVID-19 to take part. Information regarding the survey was widely disseminated across Australia by hospital leaders, professional societies, colleges, universities, associations, government health departments, and the media (using a convenience sample approach). Only doctors working in hospitals are included in this analysis, with data from general practitioners working in primary care analysed and reported separately (manuscript pending publication). Participants self-identified as senior doctors (i.e., consultant specialist) or junior doctors (all other grades).

### 2.2. Data Collection

Participants provided online consent to participate in the study, then accessed the online survey directly from the invitation or via a purpose-built website (https://covid-19-frontline.com.au/ (accessed on 15 December 2021)). Participants completed the survey questionnaire only once. Data were collected and managed using REDCap electronic data capture tools [29]. Information collected included demographics, professional background and workplace, the impact of the pandemic on employment and finances, organisational leadership and workplace change, health and recreational habits, self-reported mental health issues (subjectively determined), and five validated, objective mental health symptom measurement tools (the Generalised Anxiety Disorder (GAD-7) for anxiety [30], Patient Health Questionnaire (PHQ-9) for depression [31], abbreviated Impact of Event Scale (IES-6) for post-traumatic stress disorder (PTSD) [32], abbreviated Maslach Burnout Inventory (MBI) for burnout, and abbreviated 2-item CD-RISC-2 scale for resilience [33]). Full survey details can be found in [6]. Ethics approval was provided by the Royal Melbourne Hospital Human Research Ethics Committee (HREC/67074/MH-2020).

### 2.3. Statistical Methods and Data Analysis

All analyses were performed using SPSS version 26.0 (IBM Corp, Armonk, NY, USA) or SAS version 9.4 (SAS Institute, Cary, NC, USA). Demographic and socioeconomic characteristics are reported descriptively. For each mental health scale, outcomes were merged into dichotomous categories as described in [6]. Categorical variables were reported as frequencies and percentages and were compared between junior and senior doctors using a chi-square test. Numerical Likert scale measures of confidence and resilience were reported as means and standard deviations and were compared using the independent samples *t*-test.

Predictors of coping strategies and help-seeking behaviour were identified separately for junior and senior doctors using univariable and multivariable logistic regression models. Variables examined in univariable analyses included: age, gender, state, lives alone, lives with children, lives with elderly people, frontline area, practice location, works with COVID-19 patients, anticipates working with COVID-19 patients, received PPE training, worried their role will lead to COVID-19 transmission to family, worried about being blamed by colleagues, close friends or relatives infected with COVID-19, changed relationships with partner or friends or family or colleagues, changed household income, concerns regarding household income and pre-existing mental health diagnoses. Multivariable models were constructed using both stepwise selection and backward elimination procedures before undergoing a final assessment for clinical and biological plausibility. Variables with a *p*-value of less than 0.10 on univariable analyses or those deemed to be clinically relevant were considered for inclusion in the multivariable models. Results from the regression models are presented as odds ratios (OR) with 95% confidence intervals (95% CI) and forest plots. A two-sided *p*-value less than 0.05 was chosen to indicate statistical significance.

## 3. Results

The data from complete responses received from 1966 hospital doctors (junior 745, 37.9%; senior 1221, 62.1%) are reported here. Both junior and senior doctors predominantly resided in Victoria and practiced in metropolitan areas in a public setting (Table 1). The majority of junior doctors worked full time (656, 88.1%) whilst senior doctors were split between full time (657, 53.8%) and part time (528, 43.2%) work. More than half (684, 56.0%) of senior doctors had children at home compared to one in five junior doctors (164, 22.0%).

### 3.1. Prevalence of Mental Health Issues

Self-reported, self-determined mental health issues since the onset of the pandemic were common in all doctors, with comparable rates of anxiety (senior 54.1% vs. junior 58.0%) and PTSD (senior 3.8% vs. junior 2.6%) in both groups. Junior doctors self-reported burnout (senior 46.2% vs. junior 57.6%, *p* < 0.001), depression (senior 17.1% vs. junior 24.8%, *p* < 0.001), and other non-specified mental health issues (senior 1.8% vs. junior 3.4%, *p* < 0.001) at higher rates than seniors. On the validated scales, the prevalence of moderate to severe symptoms of anxiety (senior 18.6% vs. junior 28.9%, *p* < 0.001), depression (senior 14.1% vs. junior 19.5%, *p* = 0.002), PTSD (senior 31.0% vs. junior 44.2%, *p* < 0.001), and burnout (emotional exhaustion: senior 61.4% vs. junior 75.7%, *p* < 0.001; depersonalisation: senior 35.7% vs. junior 56.2%, *p* < 0.001; low personal accomplishment: senior 25.4% vs. junior 30.7%, *p* = 0.011) was notable in all participants, but significantly higher in junior doctors relative to senior doctors.

### 3.2. Utilisation of Coping Strategies and Help-Seeking Behaviour

The majority of respondents reported using one or more strategies to manage their mental health during the pandemic (Table 2). Maintaining exercise was the most common strategy for both groups but was utilised significantly more by senior doctors compared to juniors (*p* < 0.001). By contrast, junior doctors were significantly more likely to use yoga and meditation (*p* < 0.001) or psychological wellbeing apps (*p* = 0.002) than their senior counterparts. Both junior and senior doctors reported maintaining or increasing social interactions with friends and family at similar rates. Approximately one quarter of respondents reported increasing alcohol use during the pandemic, which did not differ significantly between groups.

Seeking formal help for mental health concerns was uncommon for both groups, with more than three quarters using no support services. Junior doctors were significantly more likely than senior doctors to see another doctor or psychologist for help with mental health symptoms during the pandemic (*p* = 0.005), albeit at low rates. Very few doctors reported seeking mental health support from employee or professional services at or outside their workplace.

### 3.3. Predictors of Coping Strategies and Help-Seeking Behaviour

**Figure 1:**
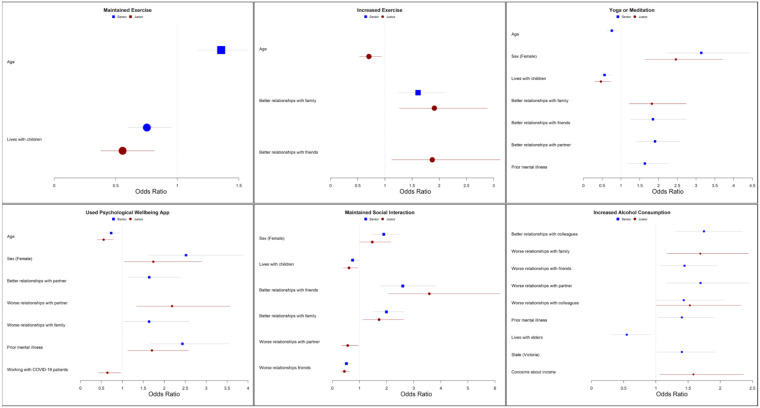
Personal and workplace predictors of coping strategies used by junior and senior medical staff. Red circles = junior, blue squares = seniors. Lines represent odds ratio and 95% confidence intervals. Reference categories: age (ordinal), female vs. male, Victoria vs. all other states, lives alone vs. with others, children vs. none, elderly care vs. none, worse relationships vs. neutral, better relationships vs. neutral, prior mental health diagnosis vs. none, concerns about income vs. negative response, family or friend infected with COVID-19 vs. negative response.

In the separate multivariable regression analyses for junior and senior doctors, differential predictors for coping and help-seeking were identified. For senior doctors, significant independent predictors of using yoga or meditation as a coping strategy included: female sex (*p* < 0.001), improved relationships with partner (*p* < 0.001) or friends (*p* = 0.002) and having prior mental health diagnoses (*p* = 0.004). For junior doctors, independent predictors of using yoga or meditation included female sex (*p* < 0.001) and improved relationships with family (*p* = 0.004). By contrast, senior and junior doctors with children at home were less likely to engage in yoga or meditation (*p* < 0.001). Other significant, independent predictors for different coping strategies for senior and junior doctors are indicated in Figure 1 and Table S1.

**Figure 2:**
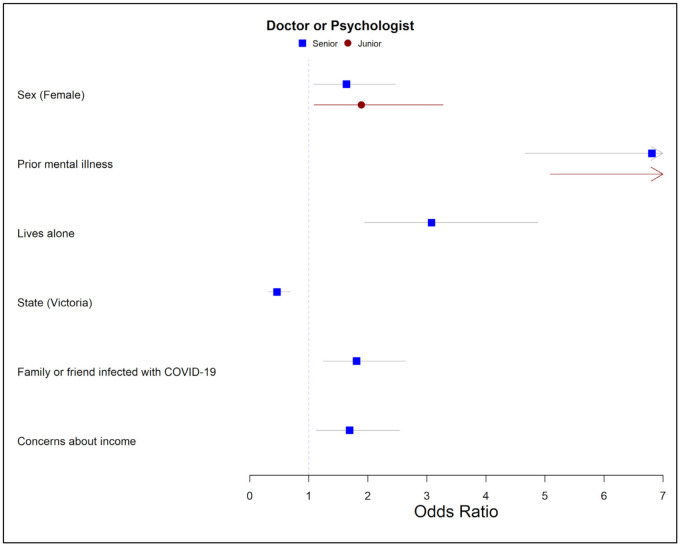
Personal and workplace predictors of seeking help from a doctor or psychologist for junior and senior medical staff. Red circle = junior, blue square = senior. Lines represent odds ratio and 95% confidence intervals. Reference categories: female vs. male, prior mental illness vs. none, lives alone vs. with others, Victoria vs. other states, family or friend infected with COVID-19 vs. negative response, concerns about income vs. negative response.

Independent predictors for seeking help from a doctor or psychologist for senior doctors included: female sex (*p* = 0.018), living in Victoria (*p* < 0.001), living alone (*p* < 0.001), having a family member or friend infected with COVID-19 (*p* = 0.002), having concerns about household income (*p* = 0.011), and prior mental health diagnosis (*p* < 0.001) (Figure 2 and Table S2). Independent predictors of seeking help from a doctor or psychologist for junior doctors included female sex (*p* = 0.023) and having a prior mental health diagnosis (*p* < 0.001).

### 3.4. Coping Strategies, Help-Seeking Behaviour and Mental Health Symptoms

Significant associations were detected between several mental health symptoms and utilisation of coping strategies (Table 3). For both junior and senior staff, moderate to severe symptoms of anxiety were significantly associated with not maintaining social interactions (senior *p* = 0.008, junior *p* = 0.015) and increased alcohol consumption (*p* < 0.001 for both). Moderate to severe symptoms of anxiety in senior doctors were also associated with not maintaining (*p* = 0.0021) or increasing exercise (*p* = 0.013), but were associated with increased use of psychological wellbeing apps (*p* = 0.003). Moderate to severe symptoms of depression were associated with increased alcohol consumption for both senior (*p* < 0.001) and junior (*p* < 0.001) doctors. Having moderate to severe symptoms of depression were also associated with increased usage of psychological wellbeing apps for senior doctors (*p* = 0.005). For junior staff, having moderate to severe symptoms of PTSD were associated with increased use of yoga or meditation (*p* = 0.004) and psychological wellbeing apps (*p* < 0.001), as well as increased alcohol consumption (*p* < 0.001). In senior staff, moderate to severe symptoms of PTSD were associated with not maintaining exercise (*p* = 0.011), increasing use of psychological wellbeing apps (*p* = 0.013), and increased alcohol consumption (*p* < 0.001).

In both junior and senior doctors, moderate to severe symptoms of the depersonalisation subdomain of burnout were associated with not maintaining social interactions (senior *p* = 0.019, junior *p* = 0.042) and increasing alcohol consumption (*p* < 0.001 for both). In senior doctors, moderate to severe depersonalisation was additionally associated with use of psychological wellbeing apps (*p* = 0.005). Moderate to severe symptoms of the emotional exhaustion subdomain of burnout in both junior and senior doctors were associated with not maintaining social interactions (senior *p* = 0.023, junior *p* = 0.003) and increased alcohol consumption (*p* < 0.001 for both). Moderate to severe symptoms of emotional exhaustion in senior doctors were additionally associated with not increasing exercise (*p* = 0.016), as well as increasing yoga and meditation (*p* = 0.018) and using psychological wellbeing apps (*p* < 0.001). Low levels of personal achievement in both junior and senior doctors were associated with not maintaining social interactions (senior *p* = 0.023, junior *p* = 0.016). In senior doctors, low personal achievement was also associated with not maintaining exercise (*p* = 0.018).

For both junior and senior doctors, symptoms of moderate to severe anxiety, depression, and PTSD were associated with increased rates of engaging with a doctor or psychologist to manage stress and mental health relative to those with none to mild symptoms (anxiety: junior 31.2% vs. 12.6%; senior 30.0% vs. 9.5%; *p* < 0.001 for both; depression: junior 27.6% vs. 15.7%, *p* = 0.001; senior 25.6% vs. 11.3%, *p* < 0.001; PTSD: junior 26.8% vs. 10.9%; senior 22.8% vs. 9.1%; *p* < 0.001 for both). Similarly, symptoms of moderate to high burnout were associated with professional help-seeking (depersonalisation: junior 21.8% vs. 13.1%, *p* = 0.002; senior 18.0% vs. 10.7%, *p* < 0.001; emotional exhaustion: junior 21.8% vs. 6.2%; senior 18.3% vs. 5.4%, *p* < 0.001 for both). Low personal achievement was associated with increased help-seeking for senior doctors (16.6% vs. 12.2%, *p* = 0.048) but did not significantly alter junior doctors’ engagement with professional help (19.6% vs. 17.3%, *p* = 0.468).

## 4. Discussion

To our knowledge, this is the largest study to examine the coping and help-seeking behaviours adopted by junior and senior doctors working across a diverse range of frontline areas during the COVID-19 pandemic. Both junior and senior doctors reported using a variety of coping strategies to manage mental health symptoms during the pandemic. The types and predictors of coping strategies utilised varied by doctors’ seniority.

Despite the prevalence of mental health symptoms in the current survey being considerably higher than previously reported, particularly for junior doctors [20], engagement with help-seeking services was extremely low overall. This occurred despite participants recognising (as measured by responses regarding self-determined, self-reported mental health symptoms) that they were experiencing these symptoms. Experiencing moderate to severe symptoms of mental illness was broadly associated with reduced utilisation of adaptive coping strategies, such as maintaining social interactions and physical exercise, and increased use of maladaptive strategies, specifically increasing alcohol consumption.

### 4.1. Coping Strategies Varied by Seniority

The vast majority of participants reported using at least one form of coping strategy and these varied between junior and senior doctors. For both juniors and seniors, maintaining or increasing physical exercise were among the most commonly used strategies. This is consistent with studies of healthcare workers in New York, where 59% reported using physical exercise to cope during the COVID-19 pandemic [16]. Increasing exercise was also more common amongst those who reported closer relationships with friends since the onset of the pandemic and is likely reflective of exercise being one of the few permitted reasons to leave home and meet up with another person during the strictest Australian restrictions [34].

Maintaining social support was reported by one in three participants overall and did not differ between junior and senior doctors. Social support is an important mediator of mental health outcomes during a crisis. Chinese healthcare workers reporting low levels of social support in March 2020 had worse outcomes on anxiety scores and an increased likelihood of engaging in maladaptive coping strategies [17]. In the current study, those who experienced moderate to severe symptoms of anxiety or burnout were less likely to maintain social interaction. Concerningly, both junior and senior doctors with children living at home were less likely to maintain social interaction. Although the current study assessed social interaction rather than directly measuring loneliness, these results echo the finding that one in two parents of school-aged children in the UK felt lonely or isolated during the first 100 days of lockdown [35]. In the UK parents cohort, female gender was also associated with increased feelings of isolation [31], whereas women in the current study were more likely to maintain social interactions. Further work is needed to identify whether maintained social interaction is sufficient to combat loneliness or feelings of isolation in doctors.

Having children at home similarly was associated with not maintaining exercise or taking part in meditation or yoga activities in the current study and may reflect a lack of time for parents, particularly those who found themselves with an increased workload at work and in the home as many children shifted to home schooling. The use of mindfulness based coping strategies, namely yoga and meditation or psychological wellbeing apps, was significantly greater in junior doctors. Senior doctors who experienced moderate to severe symptoms of anxiety, depression, PTSD, or burnout were, however, more likely to utilise apps compared to senior doctors with no or minimal symptoms. Only PTSD was associated with increased likelihood of using apps for junior doctors. These results indicate that app usage in senior doctors was driven by symptom burden although junior doctors had a higher usage in the absence of symptoms. Women were again more likely to more likely to utilise yoga or meditation, and this is likely reflective of a demographic which already uses yoga or meditation at higher rates [36]. Further research including exploratory qualitative studies are needed to gain further insight into how men cope and facilitate the creation of targeted approaches to enable men to best engage with their preferred coping strategies during crises.

### 4.2. Low Levels of Engagement with Professional Support Services

Despite the high burden of mental health symptoms and self-reported problems, very few doctors reported engaging with psychologists or doctors for professional support regarding their mental health and wellbeing. Even fewer utilised formal support services such as workplace employee assistance programs or external doctors’ health advisory services. This likely reflects of broader cultural issues around the stigma associated with mental illness and ensuing reluctance to seek help in the general population. Stigma around help-seeking and worries about mandatory reporting affecting their ability to practice medicine if doctors reveal mental health symptoms are important barriers [21,24]. Nevertheless, a recent study by Beyond Blue identified that Australian doctors seek treatment for mental health problems at higher rates than the general public [20].

Junior doctors in our study reported slightly greater engagement with mental health support services relative to senior doctors, albeit at very low rates. By contrast, historically, junior doctors have reported higher levels of hesitancy regarding discussing mental health concerns than their senior counterparts [21]. The current findings may represent a promising cultural shift in mental health awareness and are consistent with demands from junior doctors for a healthier work-life balance [37].

Although the current study did not examine barriers to help-seeking behaviour, telehealth delivery presents a host of challenges, which may prevent help-seeking, particularly amongst older people [38,39]. Junior doctors reported more usage of psychological wellbeing apps, such as Smiling Mind, Calm, and Headspace, and are a younger demographic who may have had greater preparedness to access care via telehealth or online platforms. Senior doctors who resided in Victoria, where lockdown restrictions at the time of the survey were the most severe and prolonged, were less likely to seek formal help, further indicating that lack of face-to-face options may have been particularly limiting for senior doctors.

Among both junior and senior doctors, those with prior mental health diagnoses were more likely to utilise professional support. Prior mental health diagnoses in particular were associated with around a seven-fold increase in engaging with a doctor or psychologist to manage mental health during the pandemic. This finding is likely indicative that these people were already aware of, and utilised, mental health supports services prior to the pandemic. Female gender was also associated with increased help-seeking behaviour, as well as an increase in maintaining social interactions as a means of coping. This is consistent with literature indicating that men are less likely to discuss mental health concerns or seek formal help, both within the medical profession [20] and in the broader population [40].

### 4.3. Increased Alcohol Consumption Associated with Symptoms of Mental Illness

The current survey did not quantify baseline or current alcohol consumption; however, the self-reported increase in alcohol consumption since the onset of the pandemic by one in four doctors is concerning. Reporting increased alcohol consumption in the current study was associated with worse outcomes on nearly all of the validated mental health scales tested. The emotional exhaustion subdomain of burnout was a particularly strong association, with junior doctors showing moderate to severe burnout symptoms reporting increased alcohol consumption at double the rate of those with minimal or no symptoms.

Although doctors generally consume harmful levels of alcohol at lower rates than the general public [41], a survey of 2999 Australian doctors in 2007 reported potentially hazardous levels of consumption in 8% of participants [42]. Additionally, a survey of the Australian general public between April and May 2020 reported increased alcohol consumption in one in five participants [43], indicating that doctors were increasing consumption at higher rates than the rest of the population. The wording in the current survey prompted participants to indicate whether they had increased alcohol consumption specifically as a means of coping. Although previous studies in the UK have indicated that similar medical cohorts do frequently use alcohol as a means of coping, with 34% reporting that alcohol made them feel better and 22% using alcohol to get through stressful times [44], the wording in the current survey may have resulted in under-reporting of alcohol consumption by those who did not specifically view it as a coping mechanism.

Both junior and senior doctors who reported household income concerns since the onset of the pandemic were more likely to increase alcohol consumption, as were those who reported worsening of relationships with colleagues. Together these indicate that increasing alcohol consumption was at least in part associated with increased stressors in the workplace as a result of COVID-19. Similarly, senior doctors who resided in Victoria or had a prior mental health diagnosis were also more likely to report increased consumption, indicating that the impacts of lockdown may have disproportionately resulted in maladaptive coping in this group. Initiatives by the Alcohol and Drug Foundation in August, 2020 have aimed to reduce alcohol consumption during the lockdown [45], though further targeted interventions may be necessary to combat increased alcohol consumption specifically in senior doctors. Although senior doctors in the current study did not report increasing consumption at a rate significantly higher than juniors, data from UK doctors prior to COVID-19 found that increased time in the profession was associated with increased frequency of consumption but reduced frequency of binge-drinking [44]. Further work is needed to differentiate specific changes in alcohol consumption as a result of the pandemic.

Failure to address potentially harmful alcohol consumption during the COVID-19 crisis may have long-term detrimental effects. A study of 549 Chinese healthcare workers in the wake of the 2003 SARS pandemic reported that those who reported drinking alcohol to cope during the crisis experienced six times higher symptoms of alcohol dependence three-years post-outbreak relative to those who did not use alcohol to cope [13]. Early data from the Australian Bureau of Statistics [46] show similar lingering effects, with 14% of the general public reporting increased alcohol consumption relative to before the pandemic, despite the majority of the country easing restriction in light of low caseloads at the time.

### 4.4. Implications

Engagement with formal support services, particularly those provided by workplaces or professional associations, are concerningly low and echo the known stigmatisation of seeking help for mental health concerns within the profession. The current study indicates that maintaining physical exercise and social interaction are important coping strategies that may help mitigate harms to mental health during extended crises. Formal supports provided to medical staff must be proactive in their outreach and be tailored to the preferred coping strategies of this group. Particularly, promotion of exercise engagement, opportunities that foster social activity and peer support programs should be prioritised. Given the number of medical staff reporting increased alcohol consumption and its association with adverse mental health impacts, targeted education and interventions around alcohol consumption are required. Further qualitative research is needed to co-design effective and acceptable support initiatives.

### 4.5. Strengths and Limitations

The survey distribution method prevents calculation of response rate. Selection bias may over- or under-represent participants reporting exposure to COVID-19 patients, changes in working conditions, and impacts on mental health and moral distress. Women doctors were over represented in this survey compared to workforce data from the Australian Health Practitioners Regulatory Agency indicating that women make up 44% of the overall medical workforce [47] and 52% of trainees [48]. Although the survey allowed free-text input of ‘other’ coping strategies, this was not extensively completed. As such, coping strategies which were not explicitly listed in the survey may not be adequately represented. Further research regarding how best to target adaptive coping strategies to doctors is required.

## 5. Conclusions

Junior and senior hospital doctors reported utilising a range of coping strategies to manage mental health during the pandemic. These strategies varied by seniority, with junior doctors opting to use technology and mindfulness-based strategies at slightly higher rates than their senior counterparts. Increasing alcohol consumption was reported by one in four participants and was broadly associated with symptoms of anxiety, depression, PTSD and burnout. Help-seeking behaviour overall was low; however, junior doctors sought professional support at higher rates than senior doctors and at a level consistent with the broader Australian healthcare worker population. These findings may be utilised to tailor support resources to the preferences of junior and senior doctors.

## Figures and Tables

**Table 1:** Doctors’ characteristics.

Characteristic	Senior (*n* = 1221)		Junior (*n* = 745)	
	*n*	%	*n*	%
**Age (years)**				
20–30	3	0.2	361	48.5
31–40	325	26.6	339	45.5
41–50	483	39.6	38	5.1
50+	410	33.6	7	0.9
**Sex**				
Male	498	40.8	203	27.2
Female	715	58.6	536	71.9
Non-binary	5	0.4	1	0.1
Prefer not to say	3	0.2	5	0.7
**State**				
Victoria	932	76.3	613	82.3
Other states	289	23.6	132	17.8
**Location of practice**				
Metropolitan	1068	87.5	661	88.7
Regional or Remote	153	12.5	84	11.2
**Health organisation type #**				
Public	1118	91.6	735	98.7
Community	277	22.7	33	4.4
Private	470	38.5	54	7.2
Other	58	4.8	9	1.2
**Frontline area**				
Emergency Department	173	14.2	159	21.3
ICU	92	7.5	94	12.7
Anaesthetics/peri-op/surgical	261	21.4	74	9.9
Medical specialty areas *	642	52.6	391	52.5
Other **	26	2.1	15	2.0
Community ***	27	2.2	12	1.6
**Current Employment Status**				
Full time	657	53.8	656	88.1
Part time	528	43.2	63	8.5
Casual/other	36	2.9	26	3.5
**Number of people in the household**				
Lives alone	145	11.9	133	17.9
Children < 16 years at home	684	56.0	164	22.0
Person aged ≥ 65 at home	109	8.9	38	5.1

# Multiple options could be selected; * includes general medicine, hospital aged care, respiratory medicine, infectious diseases & palliative care; ** includes radiology, pathology, and other medical areas; *** includes community specialty clinic and palliative care.

**Table 2:** Mental health, coping strategies, and help-seeking behaviour.

Categories	Senior (*n* = 1221)		Junior (*n* = 745)		Chi-Square
	*n*	%	*n*	%	*p*
**Pre-existing mental health condition diagnosed before the pandemic**					<0.001
No or prefer not to say	973	79.7	539	72.3	
Yes	248	20.3	206	27.7	
Activities to manage possible mental health issues since the pandemic started *					
Maintained exercise	579	47.4	275	36.9	<0.001
Increased exercise	334	27.4	179	24.0	0.103
Yoga, meditation or similar	269	22.0	219	29.4	<0.001
Maintained or increased social interaction with family and friends	352	28.8	243	32.6	0.076
Used a psychological wellbeing App (e.g., Smiling Mind, Headspace or other)	149	12.2	129	17.3	0.002
Increased alcohol use	341	27.9	188	25.2	0.191
Other strategy	168	13.8	94	12.6	0.47
None of the above	145	11.9	112	15.0	0.044
Sought help for stress or mental health issues from other sources *					
Doctor or psychologist	162	13.3	134	18.0	0.005
Employee support program at place of work	39	3.2	20	2.7	0.521
Professional support program outside of work	23	1.9	17	2.3	0.544
Other	29	2.4	30	4.0	0.037
None of the above	997	81.7	569	76.4	0.005

* multiple options could be selected.

**Table 3:** Associations between coping strategies and mental health outcome.

Mental Health Symptom		Maintained Exercise		Increased Exercise		Yoga or Meditation		Maintained Social Interactions		Used Psychological Wellbeing App		Increased Alcohol	
		No	Yes	No	Yes	No	Yes	No	Yes	No	Yes	No	Yes
**Junior**													
**Anxiety**	None-mild	61.9%	38.1%	76.6%	23.4%	71.7%	28.3%	**64.7%**	**35.3%**	83.8%	16.2%	**79.6%**	**20.4%**
	Mod-severe	66.0%	34.0%	74.4%	25.6%	67.9%	32.1%	**74.0%**	**26.0%**	80.0%	20.0%	**62.8%**	**37.2%**
	***p***	0.286		0.527		0.303		**0.015**		0.217		**<0.001**	
**Depression**	None-mild	62.3%	37.7%	75.6%	24.4%	70.8%	29.2%	66.6%	33.4%	83.0%	17.0%	**77.3%**	**22.7%**
	Mod-severe	66.2%	33.8%	77.2%	22.8%	70.3%	29.7%	71.0%	29.0%	81.4%	18.6%	**64.1%**	**35.9%**
	***p***	0.378		0.683		0.917		0.308		0.65		**0.001**	
**PTSD**	None-mild	63.0%	37.0%	77.8%	22.2%	**74.9%**	**25.1%**	65.9%	34.1%	**87.4%**	**12.6%**	**79.7%**	**20.3%**
	Mod-severe	62.8%	37.2%	73.8%	26.2%	**65.2%**	**34.8%**	69.8%	30.2%	**76.8%**	**23.2%**	**68.6%**	**31.4%**
	***p***	0.947		0.205		**0.004**		0.262		**<0.001**		**0.001**	
**MBI DP**	None-low	60.4%	39.6%	74.8%	25.2%	69.2%	30.8%	**63.6%**	**36.4%**	84.1%	15.9%	**81.6%**	**18.4%**
	Mod-high	65.5%	34.5%	77.2%	22.8%	72.1%	27.9%	**70.6%**	**29.4%**	81.6%	18.4%	**69.7%**	**30.3%**
	***p***	0.155		0.446		0.387		**0.042**		0.364		**<0.001**	
**MBI EE**	None-low	57.3%	42.7%	79.8%	20.2%	72.5%	27.5%	**58.4%**	**41.6%**	86.0%	14.0%	**87.1%**	**12.9%**
	Mod-high	65.2%	34.8%	75.0%	25.0%	70.3%	29.7%	**70.5%**	**29.5%**	81.6%	18.4%	**71.0%**	**29.0%**
	***p***	0.056		0.189		0.574		**0.003**		0.184		**<0.001**	
**MBI PA**	None-low	63.6%	36.4%	80.4%	19.6%	72.0%	28.0%	**73.8%**	**26.2%**	83.6%	16.4%	70.7%	29.3%
	Mod-high	63.2%	36.8%	74.2%	25.8%	70.3%	29.7%	**64.8%**	**35.2%**	82.3%	17.7%	76.8%	23.2%
	***p***	0.924		0.068		0.636		**0.016**		0.675		0.079	
**Senior**													
**Anxiety**	None-mild	**51.0%**	**49.0%**	**71.1%**	**28.9%**	78.0%	22.0%	**69.5%**	**30.5%**	**89.1%**	**10.9%**	**74.9%**	**25.1%**
	Mod-severe	**59.5%**	**40.5%**	**79.3%**	**20.7%**	78.0%	22.0%	**78.4%**	**21.6%**	**81.9%**	**18.1%**	**59.5%**	**40.5%**
	***p***	**0.021**		**0.013**		0.998		**0.008**		**0.003**		**<0.001**	
**Depression**	None-mild	**51.3%**	**48.7%**	71.8%	28.2%	78.6%	21.4%	70.2%	29.8%	**88.9%**	**11.1%**	**74.7%**	**25.3%**
	Mod-severe	**59.9%**	**40.1%**	77.3%	22.7%	74.4%	25.6%	76.7%	23.3%	**81.4%**	**18.6%**	**57.0%**	**43.0%**
	***p***	**0.036**		0.134		0.219		0.079		**0.005**		**<0.001**	
**PTSD**	None-mild	**49.9%**	**50.1%**	72.8%	27.2%	78.9%	21.1%	69.5%	30.5%	**89.4%**	**10.6%**	**77.5%**	**22.5%**
	Mod-severe	**57.8%**	**42.2%**	71.9%	28.1%	76.1%	23.9%	74.8%	25.2%	**84.4%**	**15.6%**	**60.7%**	**39.3%**
	***p***	**0.011**		0.734		0.279		0.059		**0.013**		**<0.001**	
**MBI DP**	None-low	50.6%	49.4%	72.2%	27.8%	79.0%	21.0%	**68.90%**	**31.1%**	**89.8%**	**10.2%**	**77.6%**	**22.4%**
	Mod-high	55.7%	44.3%	72.7%	27.3%	76.2%	23.8%	**75.3%**	**24.7%**	**84.3%**	**15.7%**	**62.4%**	**37.6%**
	***p***	0.094		0.849		0.253		**0.019**		**0.005**		**<0.001**	
**MBI EE**	None-low	52.2%	47.8%	**68.5%**	**31.5%**	**81.6%**	**18.4%**	**67.5%**	**32.5%**	**92.3%**	**7.7%**	**80.5%**	**19.5%**
	Mod-high	52.6%	47.4%	**74.9%**	**25.1%**	**75.8%**	**24.2%**	**73.5%**	**26.5%**	**85.1%**	**14.9%**	**66.9%**	**33.1%**
	***p***	0.918		**0.016**		**0.018**		**0.023**		**<0.001**		**<0.001**	
**MBI PA**	None-low	**58.3%**	**41.7%**	75.2%	24.8%	77.9%	22.1%	**76.2%**	**23.8%**	90.2%	9.8%	73.3%	26.7%
	Mod-high	**50.5%**	**49.5%**	71.5%	28.5%	78.1%	21.9%	**69.4%**	**30.6%**	87.0%	13.0%	71.8%	28.2%
	***p***	**0.018**		0.209		0.935		**0.023**		0.140		0.606	

Bold text indicated *p* ≤ 0.05. PTSD = post-traumatic stress disorder; MBI = Maslach burnout inventory. Burnout on the MBI is indicated by higher scores on the emotional exhaustion (EE) and depersonalisation (DP), and lower scores on the scale of personal achievement (PA); DP: 0–3 = low, 4–18 = moderate to high; EE: 0–6 = low, 7–18 = moderate to high; PA: 0–13 = low, 14–18 = moderate to high; PTSD: 0–9 = min/none and >10 = mod-severe on IES-6; anxiety: 0–9 = none/mild, 10–21 = moderate to severe on GAD7; depression: 0–9 = none/minimal to mild, 10–27 = moderate to severe of PHQ9.

## Data Availability

Data are available upon reasonable request to the corresponding author.

## Supplementary Materials

The following are available online at https://www.mdpi.com/article/10.3390/ijerph182413275/s1, Table S1: Personal and professional predictors of coping and help-seeking for junior doctors, Table S2: Personal and professional predictors of coping and help-seeking for senior doctors.
