# Supplementary material for: Differences in Coping Strategies and Help-Seeking Behaviours among Australian Junior and Senior Doctors during the COVID-19 Pandemic
Source: Int J Environ Res Public Health. 2021 Dec 16;18(24):13275. doi: 10.3390/ijerph182413275 (PMC8706730; doi:10.3390/ijerph182413275)
Supplement: Supplementary file 1 [file ijerph-18-13275-s001.zip › ijerph-1477977-supplementary.pdf]

Table S1: Personal and professional predictors of coping and help-seeking for junior doctors

Junior - multivariate	Maintained Exercise		Increased Exercise		Yoga or Meditation		Maintain Social Activity		Used App		Increased Alcohol		Doctor or Psychologist	
	OR (95% CI)	p	OR (95% CI)	p	OR (95% CI)	p	OR (95% CI)	p	OR (95% CI)	p	OR (95% CI)	p	OR (95% CI)	p
**Age (ordinal)**	-	NA	0.71 (0.53-0.94)	0.016	-	NA	-	NA	0.56 (0.39-0.78)	0.001	-	NA	-	NA
**Female**					2.46 (1.64-3.71)	<0.001	1.47 (1.00-2.17)	0.050	1.74 (1.04-2.90)	0.034			1.89 (1.09-3.89)	0.023
**Children**	0.56 (0.30-0.82)	0.003	-	NA	0.47 (0.30-0.72)	0.001	0.61 (0.40-0.94)	0.025	-	NA				
**Lives Alone**													-	NA
**State (Victoria)**	-	NA												
**Worse relationship with:**														
Partner							0.57 (0.33-0.96)	0.036	2.19 (1.34-3.58)	0.002	-	NA	-	NA
Family					-	NA	-	NA			1.69 (1.17-2.44)	0.005	-	NA
Friends							0.44 (0.31-0.63)	<0.001			-	NA		
Colleagues	-	NA			-	NA	-	NA			1.53 (1.01-2.32)	0.047		
**Better relationship with:**														
Partner			-	NA										
Family	-	NA	1.91 (1.27-2.88)	0.002	1.82 (1.22-2.73)	0.004	1.72 (1.13-2.63)	0.012						
Friends			1.87 (1.12-3.12)	0.016	-	NA	3.57 (2.06-6.19)	<0.001	-	NA				
Colleagues							-	NA						
**Prior Mental Health Diagnosis**									1.71 (1.13-2.58)	0.011			7.73 (5.09-11.75)	<0.001
**Concerns about household income**							-	NA			1.58 (1.06-2.36)	0.024		
**Currently working with COVID-19 patients**					-	NA			0.65 (0.43-0.96)	0.032	-	NA		
**Frontline area**	-	NA	-	NA	-	NA	-	NA	-	NA	-	NA	-	NA
**Family or friend infected with COVID-19**	-	NA									-	NA		

Reference categories: age (ordinal), female vs male, children vs none, lives alone vs with others, Victoria vs all other states, worse relationships vs neutral, better relationships vs neutral, prior mental health diagnosis vs none, concerns about income vs negative response, currently working with COVID-19 patients vs negative response, frontline area vs medical, family or friend infected with COVID-19 vs negative response. NA = not significant in the multivariate analysis.

Table S2: Personal and professional predictors of coping and help-seeking for senior doctors

Senior-multivariate	Maintained Exercise		Increased Exercise		Yoga or Meditation		Maintain Social Activity		Used App		Increased Alcohol		Doctor or Psychologist	
	OR (95% CI)	p	OR (95% CI)	p	OR (95% CI)	p	OR (95% CI)	p	OR (95% CI)	p	OR (95% CI)	p	OR (95% CI)	p
**Age (ordinal)**	1.36 (1.17-0.75)	<0.001	-	NA	0.76 (0.63-0.91)	0.004	-	NA	0.73 (0.58-0.93)	0.011	-	NA	-	NA
**Female**					3.14 (2.23-4.41)	<0.001	1.89 (1.44-2.50)	<0.001	2.52 (1.63-3.88)	<0.001			1.64 (1.09-2.47)	0.018
**State (Victoria)**											1.40 (1.02-1.93)	0.036	0.46 (0.31-0.69)	<0.001
**Regional location**													-	NA
**Lives Alone**					-	NA							3.08 (1.94-4.88)	<0.001
**Children**	0.75 (0.60-0.95)	0.017	-	NA	0.56 (0.42-0.75)	<0.001	0.74 (0.57-0.98)	0.035						
**Elderly care**											0.55 (0.32-0.92)	0.024		
**Worse relationship with:**														
Partner							-	NA			1.69 (1.17-2.44)	0.005	-	NA
Family							-	NA	1.64 (1.03-2.59)	0.036	-	NA	-	NA
Friends	-	NA					0.52 (0.37-0.72)	<0.001			1.44 (1.07-1.95)	0.016	-	NA
Colleagues					-	NA			-	NA	1.43 (1.00-2.05)	0.048	-	NA

**Better relationship with:**														
Partner			-	NA	1.91 (1.17-2.28)	<0.001	-	NA	1.64 (1.14-2.37)	0.008				
Family			-	NA	-	NA	1.99 (1.50-2.65)	<0.001	-	NA				
Friends			1.61 (1.24-2.08)	<0.001	1.85 (1.25-2.75)	0.002	2.60 (1.76-3.82)	<0.001	-	NA			-	NA
Colleagues											1.75 (1.30-2.34)	<0.001		
**Prior Mental Health Diagnosis**					1.63 (1.17-2.28)	0.004	-	NA	2.43 (1.67-3.55)	<0.001	1.40 (1.03-1.91)	0.03	6.81 (4.66-9.95)	<0.001
**Concerns about household income**													1.69 (1.13-2.54)	0.011
**Currently working with COVID-19 patients**							-	NA			-	NA		
**Frontline area**	-	NA	-	NA	-	NA	-	NA	-	NA	-	NA	-	NA
**Family or friend infected with COVID-19**					-	NA			-	NA			1.81 (1.24-2.64)	0.002

Reference categories: age (ordinal), female vs male, Victoria vs all other states, regional vs metropolitan, lives alone vs with others, children vs none, elderly care vs none, worse relationships vs neutral, better relationships vs neutral, prior mental health diagnosis vs none, concerns about income vs negative response, currently working with COVID-19 patients vs negative response, frontline area vs medical, family or friend infected with COVID-19 vs negative response. NA = not significant in the multivariate analysis.
